# Medical Tour in Italy

**Published:** 1827-01

**Authors:** 


*Voyage en Italie, fait en l'Année,* 1820. *Deuxième Edition, corrigée et augmentée de Nouvelles Observations faites dans un Second Voyage en* 1824. Par le Docteur LOUIS VALENTIN, Chevalier des Ordres, &c. &c.

*Dr. Louis Valentin's Travels in Italy, in the years* 1820, *and* 1824. *Second Edition.*

"MEN run to and fro, and knowledge is increased." So says Holy Writ. But others have said—"multum mentitur qui multum vidit;" and this we fear is too true. As far as our profession is concerned, the general impression is, that the travels of a well-informed physician may prove very advantageous to his native country. This must have been the impression on Dr. Radcliffe, when he bequeathed 500 pounds per annum for the support of a medical traveller-ship—and the impression has been kept up, of course, by the treasures of medical lore, which have been poured into this country quinquennially from the budgets of the "professors of travelling," thus fed and clad by the pious bequest of the Oxonian patriot. Whether a fixed salary for doing a certain duty be a better stimulus towards its performance, than the hope of gain or fame to be acquired by individual exertion, it is not for us to determine. Neither is it our intention to institute any comparison between

---

"* Vol. i. Essay, 2. p. 29."

the products of the "travelling fellows of Oxford," and of their "fellow travellers," in the army, navy, and private life. Certain it is, that the labours of all these classes have met a favourable reception from the public, notwithstanding the "multum mentitur" reservation which must be made in perusing the narratives of medical as well as other perigrinators.

When Dr. Valentin published his first edition, under the title of "*Voyage Medicale*," he was well received, and he was stimulated to make a second journey through "le plus beau pays de l'Europe," for the purpose of enlarging and improving his first work. We confess that we should have been much better pleased, if he had adhered more to the *medical* part of his plan, and attempted less in the way of scientific researches, for which there were plenty of other tourists, at least as well qualified as himself. Dr. V. however, is ambitious of being considered a *sçavans*, and possibly, on this account, he may rather lose than gain in the character of physician.

Three leading motives determined our traveller to cross the Alps—the *first* was his HEALTH—the *second* was "a new doctrine in medicine which, for some years, attracted attention, while another in France, had acquired extraordinary celebrity"—the *third* was rather a curious motive—"the eruption of Vesuvius" "L'Eruption du Vesuve." Vulcan had set all his forges at work, in the winter of 1819, and continued his subterranean operations in April, 1820—the temptation could no longer be resisted, and the doctor set out to contemplate this grand phenomenon, and to view the skeletons of towns that had lately been disinterred from those graves of Lava, in which they had slumbered for seventeen hundred years. Dr. V. had travelled before. He had traversed the Atlantic, and spent eight years in America. He had crossed a more formidable ocean—the British Channel, and visited England and Ireland—he had even been beyond the Rhine—"au-delà du Rhin"—and, therefore, he did not go an unfledged youth into "Latium's velvet ground," but as an experienced traveller, rich in the knowledge and information which he had gleaned in various climes.

Et mores hominum multorum vidit et urbes.

Dr. Valentin felicitates the English nation on the great advantages that result from "the travelling fellowship," and seems to cast a longing eye on the "12,400 francs de nôtre monnaie," which those *happy fellows* annually pocket, for so delightful an occupation. "On conçoit qu' une pareille institution ne peut avoir que les plus heureux résultats." He would be quite in raptures, if he knew the extent of advantages which have resulted from this munificent institution. But, perhaps, in this case, as in many others, "ignorance is bliss;" and, therefore, we shall not make him acquainted with the whole of our felicity.

"The traveller," says our author, "who means to benefit by his peregrinations, should make up his mind for all contingencies, and expect to meet with many difficulties which may put his zeal and patience to trial. It is true that, marching under the banner of the God of Epidaurus, he will have many facilities, and meet with various opportunities of doing good—so true is the expression of an Emperor—'that in all places, the practice of medicine is the exercise of every virtue.'"

"No sooner does the young physician quit his native soil, than every thing around him acquires a new interest in his eyes. His soul is elevated—and his imagination enlarged. By degrees, he loses his prejudices, his intolerance, and his presumption, the constant attendants on ignorance. Perceiving the futility of those systems in which he has been educated, he finds, at last, that the learning on which he so much prided himself, becomes reduced to a very small amount. By comparing men, opinions, and things, he learns, at length, to estimate each at its just value. Nevertheless, in order that medicine and philosophy may be gainers by his travels, he should take truth for his guide—have an honest heart—kind and benevolent dispositions—judgment to resist seductive but erroneous theories—and, finally, he should cull from the nations which he visits, only their virtues and their knowledge."—*Pref. p.* 10-11.

The foregoing extract from the preface cannot fail to place our author in a favourable point of view; and, to say the truth, he seems to have taken as small a proportion of pride and prejudice over the Alps with him, as could well be expected. He appears to have tasted the bitter cup of adversity, in a long expatriation, during the terrors of the Revolution, and to have returned to his native soil full of that sentiment so often felt by Britons:—"*The more I see of other countries, the better I love my own.*"

But it is time to commence our Journey, which we do in very good humour with our agreeable companion.

Dr. V. embarked at Marseilles, and in ten days arrived in the Bay of Naples. We do not wonder at his admiration of the magnificent scenery which Vesuvius, Mount Pausilippus, Ischia, Procida, Caprea, Naples itself, and a hundred other objects combine to form. Our author hastened to climb to the crater of Vesuvius, and no doubt fancied himself a second Pliny, while contemplating the terrific heavings of old Vulcan in this, his fiery laboratory. But Dr. V. had nearly suffered the fate of the philosopher of Parthenope, for his curiosity. He was saluted by a sulphureous vapour, which instantly deprived him of the power of breathing, and his guide fortunately succeeded in dragging him down into a cooler air, where he soon recovered.

From this dangerous excursion he descended to the more tranquil and not less interesting ruins of Pompeia and Herculaneum, where he trode the identical halls and apartments once inhabited by a Sallust, a Polybius, and a Cicero. We dare not tarry with Dr. V. in these places; and we reiterate our opinion, that he might have employed 50 pages of his work to much more advantage in the investigation of the medical topography of Naples, to which city so many invalids resort for health, than in decriptions of ruins and other antiquities, which have been described over and over again by so many travellers.

### Hospitals.

The Hôpital "*des Incurables*" is a vast edifice in the centre of the city, and contained 900 patients when our author was there. Among the *incurables* admitted into this hospital, are pregnant women, "who wish to conceal the fruits of their amours." We suppose they are received under the impression that they are *incurable* in a moral rather than in a physical point of view. This supposition is strengthened by the calculation made by Cottigno, that 950 in every 1000 of the inhabitants of Naples, are afflicted with syphilis! We hope this information will induce some of our London philanthropists, as the Eadys, the Catons, and other "reformers of the constitution," to emigrate to a scene where their talents may have ample scope.

Dr. Ruggiero informed our author that phthisis claims a fifth of the bills of mortality in Naples. If this be a fact, we have little reason to declaim against the gloomy skies of old England, as far as regards pulmonary consumption. Pneumonia is rarely treated by venesection in Naples. Small doses of tartarised antimony, and afterwards digitalis and nitre, are the remedies on which they rely. The mortality in this class of complaints, varies from 4 to 8 per cent, which certainly is a testimony favourable to the practice pursued. Hæmoptysis is treated by digitalis, and so is mania sometimes. In the case of an officer recovering from this last disease, the dose of digitalis had been carried to 18 grains per diem. Dr. Savaresi informed our author, that when he first went to Martinique, he tried the antiphlogistic plan of treatment in yellow fever—and lost all his patients. He then had recourse to *tonics*—the practice of

Jackson—and was universally successful! Drs. V. and S. must have read the works of Dr. Jackson to great advantage!

The picture which Dr. V. draws of the Lunatic Hospitals is horrible, and we shall pass it over. Here our author met with the venerable Scarpa, who, at the age of 71, was travelling to see Italy, which was, he said, comparatively a *terra incognita* to him.

### STATE OF MEDICINE.

Brunonianism reigned triumphant for the space of 30 years in Naples, but is now superseded by the doctrine of *contro-stimulus*—at least, among all the young medical men, and the conductors of Journals. The older practitioners adopt an eclectic medium. Dr. Savaresi informed our author that he trusted to nothing but, "*ratio, observatio, et experientia.*" He is right. Naples has been a prey to epidemic fevers, of late years, which have been treated in various ways—sometimes on the most contrary plans. The best-informed practitioners are of opinion that the epidemics varied so much that the principles and practice of one year formed no guide for the next. This, we believe, is the case, and will be so till the end of time.

In continued fevers, the Neapolitan physicians use acidulated drink—small doses of tartarized antimony—cold affusions—ices—and blisters. The quinine is in high reputation there for the cure of intermittent, remittent, and pernicious fevers, which are very common about Capua, Salernum, and Pœstum. Auscultation had made but little progress—and local bleeding was much neglected. Lithotomy is very successful at Naples, and symphysiotomy has been four times performed with success by Dr. Bruno.

In the kingdom of Naples, as well as in Sicily, ices are in constant use by the inhabitants in the hot season, and especially during the Sirocco winds. It would be difficult, our author thinks, to live without such a necessary beverage in such times and such places. The ice gives tone to the organs of digestion, and, by its cooling properties, prevents inflammatory affections of the mucous membrane.

Phthisis, as every one knows, is considered to be highly contagious in Italy and Sicily. Bronchocele is very common at Naples, especially among females. In a French family there, the whole of the girls were affected, while the boys escaped. Quadri and his followers have abandoned the use of the seton in bronchocele. Iodine is entirely trusted to for its cure. Vaccination is unquestioned, as to its preventive powers in Naples. Those who neglect to have their children vaccinated are deprived of many privileges, and cannot receive any honours, titles, or marks of distinction from the Government.

Several medical journals are published in Naples, and Professor Lanza, whose work has been lately translated into English, is a leading character among the medical literati of the day. Mineral waters are plentiful in the environs of Naples, and some of them are powerfully impregnated with sulphuretted hydrogen gas, and, consequently, are much resorted to for the cure of cutaneous complaints.

Our author was too curious a traveller not to visit the Grotto del Cani. He found that the stratum of carbonic acid gas on the floor was about 12 inches thick. A little dog was asphyxied in our author's presence, and restored by a plunge in the neighbouring lake. Dr. V. lay down on his face, in order to put himself on a level with the dog, but the gas had no other effect on him than that of producing an acid taste in the mouth. The Doctor did not, therefore, require immersion in the lake. This is all the medical intelligence we could glean from the Neapolitan part of the tour.

### Rome.

A run of 48 leagues through Terracina, the Pontine fens, &c. over an excellent causeway, bordered, for 20 or 30 miles, with two rows of trees, brought our author to the gates of the Eternal City. Rome presents a great contrast to Naples. In the *latter*, the eye is delighted at every step—it is the land of pleasures. In Rome, every thing is sombre and tranquil—it is the land of antiquities, of wonders, and of study. We shall not follow Dr. V. in his description of architectural ruins, which he had much better have passed unnoticed.

### Hospitals.

There are eight civil and one military hospital in Rome. St. Esprit is the principal, and may contain, on an emergency, eight or nine hundred sick.

Every one has heard of the insalubrity of Rome in the summer season, and the *malaria* with which it is infested. It is still maintained by a party, (though in the minority) that the insalubrity of Rome is owing to the cutting down of the forests, (the nemora sacra of the ancients) situated between the Pontine fens and the city. This was the opinion of Lancisi. But the opposite opinion is better supported. If the fenny emanations were capable of being transported so far, without change, by the S.W. winds, how is it, ask the anti-Lancisians, that the towns of Velletri, Gensano, Albano, aud Ariccia, which are in the direct tract, should be more healthy than Rome itself? We are not inclined to partake of Dr. Valentin's opinion, that the cause of Roman insalubrity is to be sought in the neglect of public and private hygiene, of medical police, and of moral and physical education. These are very unlikely to be the causes of the annual visitations of fever in Rome. There can be little doubt that they are owing to a local febrific miasm, though we cannot account for its generation or extrication, no more than we can account for the eruptions of Mount Vesuvius, whose subterranean thunders were silent for two hundred years, and then broke forth again, without any ostensible reason. To the soil of Rome, then—to its hot and humid atmosphere—to the influence of inundations of the Tiber—to the dirtiness of the alleys and courts, are we to look for the causes of its insalubrity.

A French physician, Dr. Gonel, resident at Rome, conceives that the malaria is principally, if not solely, composed of carburetted hydrogen gas, which, he says, issues in such abundance from the earth, at certain times, and in certain parts of Rome, that it takes fire spontaneously. A great deal of sulphuretted hydrogen gas is also extricated, and proves extremely ungrateful to the respiratory organs and nerves of sense. The basin, indeed, in which the everlasting city is built, appears to have been an extinguished crater of some volcano, and the number of other extinct volcanos dispersed over the country is great.

Rome, then, being situated in a low plain, surrounded by hills, and with a Sirocco prevailing the greater part of the year, which wind is there both hot and humid, we need hardly wonder that the air should be unwholesome, especially when we reflect on the extrication of gazeous exhalations from the bowels of the earth, the choked up canals, and the dirty streets. To these may be added, the rapid and great atmospheric transitions which are so very common at Rome—the nights being generally cold, and the heat of the day intense—the dews heavy, and the mornings raw. Such a combination of circumstances is well calculated to produce fevers of every type—especially the intermittent and remittent classes. No idea is entertained that these fevers are contagious. In this respect, they assimilate with paludal fevers in other countries.

A French physician, M. Bailly, who sojourned a good while at Rome, has investigated the post-mortem appearances in these fevers, and found traces of gastro-enteritis in almost all of them. On this subject we need not at present make any remarks—except that he found the same appearances in those who died of pulmonic inflammation. In fact, we believe that few die of any disease without suffering more or less of inflammatory irritation in the mucous membrane of the bowels. Diarrhœa is one of the most frequent of all harbingers of death—but the state of membrane by which this symptom is produced appears to be a consequence, far more frequently than a cause of the fever or other disease towards the close of which it is developed.

Goitre is very little seen at Rome; and ophthalmia much less frequent than at Naples. Consumption is common as at Naples; but the creed of contagion, as respects phthisis, is on the wane in the states of the Pope.

In almost all diseases, bleeding is the first remedy among the Roman physicians; and it is considered as highly preventive of the impression of the malaria. Emetics are little employed. Bleeding is resorted to by all classes; and although some strong protests have lately been made against the practice by some writers, they are not at all attended to.* We should suspect that a custom which has taken such deep root in and out of the profession in Rome or any other country, is not likely to be void of some solid foundation. The stethoscope is neglected by the Romans, although introduced to their notice by our countryman, Dr. James Clark. Ices, as at Naples, are considered equally salutary and luxurious.

An immense expenditure of cinchona is made in Rome. Its administration is generally preceded by venesection. It is calculated that in Rome and the immediate neighbourhood, ten thousand two hundred pounds (12 ounces to the pound) weight of bark are annually consumed! The sulphate of quinine, however, is now universally adopted there. Dr. V. was assured that the celebrated *aqua tofana* was nothing more or less than a dilute solution of arsenic in water. Given slowly, it slowly occasioned death, without leaving any traces of its effects in the dead body. Vaccination languishes at Rome, being left optional with families. The population of the eternal city is rather under 148,000 souls—a great falling off since the Augustan age!† In 1819, the births were 4299, and the deaths 6114. The deaths are as 1 in 144 of the population annually. Notwithstanding that the deaths exceed the births, the population is on the increase—no doubt from the influx of strangers since the peace.

---

* The new Italian doctrine of contro-stimulus has made little progress in the Papal dominions. The doctrine of Broussais is known, but not much credited. The "médécine Hippocratique" is in most request at Rome.—*Rev.*

† Tacitus, the faithful historian, and the intimate friend of Pliny the younger, informs us that, in the reign of Claudius, the population of Rome and suburbs was nearly *seven millions*—more than six times the population of London!

### FLORENCE.

Our author travelled from Rome to Florence through the Appennines, by way of Terni, Spoletto, Cortona, and Arezzo. From Terni he went to visit the famous waterfall of Velino, 700 feet in height. But, as it is twice interrupted in this descent, it cannot be compared with the Staubach, in the romantic valley of Lauterbrunen, which falls 800 feet without a single interruption. When the waters are free, this last is the most magnificent cascade we have ever seen in any part of the world.

Florence, the great resort of the English, contains 82,000 inhabitants. It is well built, and traversed by the beautiful river Arno. In the evenings, the delightful promenade of the Cassina presents a brilliant assemblage of beauty and fashion. The affability of the Florentines, the mildness of their government, the good society, the number of public amusements, the cultivation of the arts and sciences, the salubrity and temperature of the climate, and the cheap living, have all combined to render Florence the abode of numerous families, especially English, whose *res angusta domi*, the education of their children, or their own health, compel them to emigrate for a time to a foreign land. It is to be recollected, however, that the proximity of Florence to the Appennines renders the air very piercing at certain seasons, and, therefore, not so well adapted for the pulmonary invalid as some other parts of Italy. We shall not follow Dr. Valentin's long and uninteresting detail of the wards, kitchens, and medical officers of the hospitals. Of what possible interest can such catalogues be to ninety-nine in the hundred of his readers.

Dr. Polidori appears to be one of the principal physicians of Florence. He believes that all fevers are primarily of the same nature, as to proximate cause; they only vary in consequence of difference of constitutions. The inflammations which we find in the dead body, are effects or complications, not causes of the fever. He is inclined to the humoral pathology. His method of treatment is very simple. He does not carry blood-letting to extremes. He gives emetics in gastric and peripneumonic affections. Nux vomica in paralysis, and prussic acid in pectoral complaints, have entirely failed in his hands. He treats with almost unvaried success, the most obstinate intermittents, by a mixture of twenty grains of emetic tartar and an ounce of powdered cinchona. This, we suppose, is the quantity for an intermission. Syphilis is successfully treated by mercurial frictions.

Dr. Bigeschi, of the lying-in-hospital, administers the secale cornutum, with great success, in the acceleration of parturition. He informed our author that, in more than 60 cases in which he exhibited the medicine, only two failed to shew the desired effect of the remedy. He gives 30 grains for a dose. Sometimes he gives a second dose of 20 or 24 grains, half an hour after the first. Fifty grains have produced no bad effect. Subsequent experience has confirmed Dr. B. in his opinions of this medicine.

The state of medical practice at Florence savours of the eclectic, partaking of the new Italian doctrine of contr.-stimulus, and the doctrine of Broussais. Blood-letting, local and general, is employed by all.

Our author, after filling scores of pages with accounts of observatories, cabinets of curiosities, &c. sets out and visits Sienna. The climate of this place is said to be temperate, but subject to sudden vicissitudes. The town is exposed to the mistral, or northerly wind, which is sometimes very keen. Inflammatory diseases and marsh fevers are common there.

Our author gives some particulars of the life of Mascagni, the celebrated anatomist, who was a native of this place. He was successful in the employment of large doses of sub-carbonate of potash and also of soda in calculous complaints. He died of a marsh fever, in 1815, aged about 61 years. He was of small stature, and much addicted to opium, of which he took more than 140 grains per diem. This medicine, which composes some people, and makes others very ill, had an exhilarating effect on Mascagni, and enabled him to pursue his midnight studies without fatigue. He contracted this dangerous habit when he was employed on his work on the lymphatics, and was never able to leave it off afterwards.

### Leghorn.

This fine sea-port contains about 75 thousand inhabitants. Pulmonary affections, phthisis, dysentery, gastro-enterites, intermittents, and ophthalmia prevail there. Dr. Palloni calculates that one death in five is caused by phthisis. Drs. Dufour and Martolini begin the cure of intermittent fevers, without any preparation, by giving one grain of tartar emetic and three drachms of bark, and repeat the dose twice in the course of the intermission. In bad cases the dose is increased. The sulphate of quinine is now superseding the above plan.

Leghorn, being situated on a plain, which was formerly a marsh, and close to the feet of high mountains, with much stagnant water in the fosses, contains within itself the materials for fevers of the paludal character. The temperature of the air is agreeable here, and the heat never excessive. Dr. Valentin having got on his favourite subject of yellow fever, (he is an ultra non-contagionist) we are obliged to leave him in the midst of his host of proofs that this disease is of home-growth at Leghorn, as well as at most other places.

### PISA.

This city is situated pleasantly about six miles from the sea. The population is about 16,000 souls. Formerly it amounted to 150,000! M. Vacca Berlingheri still continues to practise the recto-vesical lithotomy; and, it is said, with success.

### BOLOGNA.

This city is one of the most interesting, in a medical point of view, in Italy. It contains 80,000 inhabitants. It is here that Tommasini lives, and the new doctrine of contro-stimulus reigns. Rassori, it is true, is the founder of the new doctrine; but Tommasini has greatly modified it. The French and Italian professors both claim the important discovery, that fever is never idiopathic, but always symptomatic of some local inflammation. As we do not admit the truth of either of their doctrines, we shall not stop with M. Valentin to investigate their claims to propriety.

### PADUA.

This university is a formidable rival to that of Bologna. Padua has given birth to great men in our profession. Morgagni, Fabricius ab aquapendente Scarpa, Brera, and others, have thrown a lustre over its annals. The immortal Harvey studied there.

### VENICE.

We shall not stop long at this ancient mistress of the seas. A woman was shewn to Dr. V. by Dr. Piccoli and Dr. Baldini, who, after a sudden suppression of the menses, in consequence of a fright, became affected with discharges of blood from almost every outlet of the body. She first had hæmoptysis, then hæmorrhages from the stomach, mouth, eyelids, nipples, nose, rectum, &c. The Italian physicians were foiled, and asked Dr. Valentin's advice. He recommended them to leave off all internal emmenagogues, and to give ices, using every possible means externally of drawing blood towards the uterus, as semicupia, leeches to the vagina, &c. He had since learnt that the menses returned, and that the woman is cured.

### Milan.

This city, which is called the Paris of Italy, contains about 130,000 inhabitants. Intermittent fevers prevail here, together with pulmonic inflammations, and phthisis. Scrofula and the pellagra are common at Milan and in the plains of Lombardy. The method of Grannini, which consists in the affusion of cold water on the body, after the hot stage has become developed, is going out of use. We wonder how it ever could have come into practice. The physicians of Milan are divided into three sects—the eclectics, the contro-stimulists, and the followers of Broussais. In the first class are the medical officers of hospitals—in the second, the pupils and the partisans of Rassori. This last professor had been a most zealous Brunonian, and had translated Brown's Elements into Italian with notes; but shocked and grieved by the havoc which this doctrine had caused in the epidemic fever of Genoa, he abandoned Brunonianism, and erected on its ruins the celebrated contro-stimulism of the present day. Rassori now gives large doses of sulphate of quinine, generous wines, and opium in fevers—and swears they are excellent contro-stimulants!

He believes in the existence of idiopathic fevers—and considers the local inflammations which we find, after death, as the effects rather than the causes of the fever. He bleeds where much excitement appears in the beginning. In pulmonic and other visceral inflammations he depends chiefly on large doses of emetic tartar, with drastic purgatives. The general opinion in Italy is, that the doctrine of Rassori will shortly undergo the fate of Brunonianism. The followers of Broussais, composing the third class, are not numerous, but they are very zealous. They have a medical journal in their service.

Dr. Valentin gives some account of that curious cutaneous disease, the PELLAGRA, endemic in Lombardy. The backs of the hands and of the feet are the principal seats of the disease. The skin becomes red, wrinkled, and at last furfuraceous, but without heat or pain. The patients are emaciated, melancholy, and hypochondriacal, in a high degree. They suffer much along the spinal column, and are extremely weak, The disease becomes complicated with insanity, maniacal delirium, or pneumonia, tabes, dysentery, œdema, &c. but without any diminution of the pellagra itself. Many instances of suicide are the result of this strange malady.

This disease appears in the spring, increases in summer, and declines in the autumn. The causes are unknown, and the cure is chiefly effected by repose, milk, good diet, wine, and warm baths. Emigration to another part of the country cures it, if early put in practice.

Dr. Strambio has minutely examined 38 bodies dead of this disease, and found organic lesions in the head, the vertebral canal, the chest—but especially in the abdomen. "He was the first to demonstrate that the cutaneous affection was merely symptomatic, and that the stomach, liver, and intestines, were the primary seats of the disease." M. Brera avers that he has found the ganglionic nerves in a morbid state in this disease, which is not improbable. We wish he had given the details of his dissections.

We must now hasten to a close: under the head of Parma, where our author gives, as usual, a description of the hospitals and all other public places, we find the following case of rupture of the uterus and gastrotomy.

*Case.* Angiola Grossi, aged 44 years, had borne five children, and was pregnant of the sixth. Labour came on at the proper time. She suddenly turned sick, and fainted. When she recovered, she felt extreme pain in the abdomen, with dreadful sense of distention. The abdomen swelled—the vomitings increased—the difficulty of breathing became alarming, and Professor Jos. Rossi was summoned, who soon recognized rupture of the uterus, and the escape of the fœtus into the cavity of the abdomen. Several medical men were now summoned, and Professor Cecconi undertook the operation. This was performed two hours after the rupture of the uterus. The incision was made in the left side—the fœtus seized by the feet, and extracted, together with the placenta. The wound was sewed up, and in 40 days afterwards, Grossi was able to walk about quite well. P. 331.

Three years afterwards this woman again became pregnant, and was delivered without accident.

At length Dr. Valentin turns his face homewards, and makes various excursions among the Alps. He visited Chamouni, the Montanvert, Mere de Glace, &c. His health was now greatly improved, and he had lost many troublesome symptoms, especially palpitations of the heart, and vertigo. "Ma santé s'est trouvé meilleure; des palpitations et des vertiges, qui compliquaient mes anciennes infirmités, ont desparu."* Dr. V. cannot be very young, since he informs us that 36 years ago a medal was given him by the Royal Academy of Surgery, for an Essay on Goitre and Cretinism. He means to publish again on the same subjects.

---

* See Dr. Johnson's remarks on the effects of travelling among the Alps on the functions of the heart and of various organs, at pages 121-7 of his Essay on MORBID SENSIBILITY of the Stomach and Bowels,

We cannot help reiterating our opinion that, on such a journey as this, Dr. V. might have made a much greater number of original observations than he has done. The work may be useful, as a "compagnion de voyage," for the medical traveller; but is worth little to those who must travel by the fireside through the medium of others. We have given the whole of the purely medical information in a single sheet of our Journal.

